# Incorporating Photoresponses into Porous Liquids

**DOI:** 10.1002/chem.202303593

**Published:** 2024-01-11

**Authors:** Michael C. Brand, Hamish G. Trowell, Matthew J. Fuchter, Rebecca L. Greenaway

**Affiliations:** ^1^ Department of Chemistry, Molecular Sciences Research Hub Imperial College London 82 Wood Lane London W12 0BZ UK; ^2^ Department of Chemistry, Materials Innovation Factory and Leverhulme Research Centre for Functional Materials Design University of Liverpool 51 Oxford Street Liverpool L7 3NY UK

**Keywords:** photoswitch, porous liquids, photoresponsive, porosity

## Abstract

Porous liquids combine the properties of a porous solid with those of a liquid, creating a porous flowable media. Since their discovery, these materials have gathered widespread interest within the scientific community, with substantial numbers of new systems being discovered, often with a focus on increasing the pore volume and gas capacity. Which begs the question, what does the future hold for porous liquids? Recently, the first examples of photoresponsive porous liquids have emerged, allowing changes in porosity to be observed under UV irradiation. Here, we expand on our previous report of photoresponsive porous liquids and explore the conceptualisation of responsive porous liquids and how these materials could be developed with the ability to respond to light, thereby offering a potential mechanism of controllable uptake and release in these systems. This concept article summarises different approaches that could be used to incorporate a photoresponse in a porous liquid before discussing recently reported systems, alongside important factors to consider in their design. Finally, by taking inspiration from the methods used to translate porous solids into the liquid state, combined with the field of photoresponsive materials, we discuss potential strategies that could be employed to realise further examples of photoresponsive porous liquids.

## Graphical abstract

Porous liquids are a relatively new class of porous material, and while a large proportion of research has been aimed at improving gas capacity and selectivity, there has been less focus on the selective and controllable release of gas from within the material. With a handful of examples now reported, we discuss the concept of photoresponsive porous liquids, aimed at invoking a change in the gas capacity on irradiation with light, and discuss important factors in their design, alongside potential strategies for the realisation of further systems.

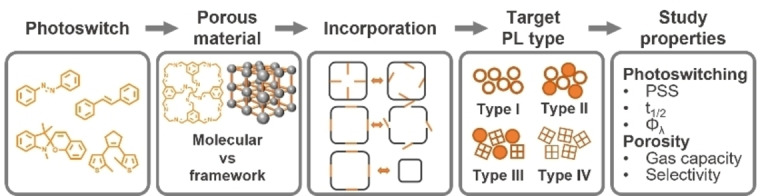

## Introduction

**Figure 1:**
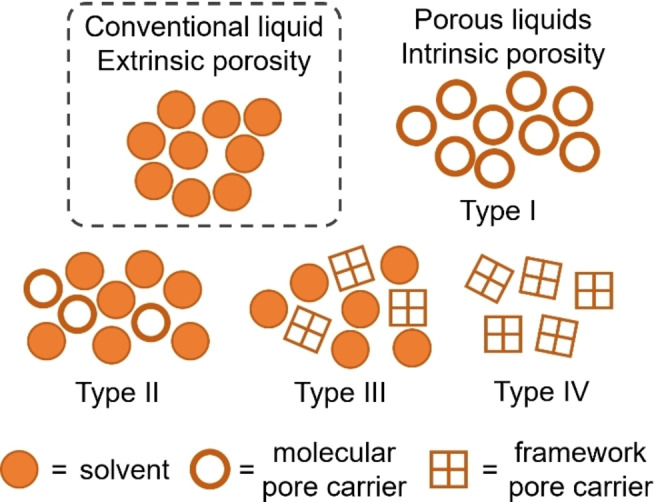
Schematic of conventional liquids, which only contain extrinsic porosity, and porous liquids which contain permanent intrinsic porosity as four types: type I – neat molecular liquids consisting of porous hosts; type II – pore carrier dissolved in a cavity‐excluded solvent; type III – pore carrier dispersed in a cavity‐excluded solvent; and type IV – porous meltable frameworks.

Porous materials have been at the forefront of materials discovery over the past 30 years due to their applications in areas such as catalysis, gas storage and separations, and energy storage. Until recently, these materials have typically been solid in nature, which is arguably their biggest limitation. However, in 2007, James and co‐workers proposed a new type of porous material which was conceptualised as ‘porous liquids’ or liquids with permanent porosity.^[1]^ Conventional liquids can display porosity to some extent, due to the transient formation of extrinsic pores which arise from the disorder and dynamic structure of liquids.^[2]^ Porous liquids (PLs) differ from conventional liquids as they contain permanent intrinsic and accessible cavities. Four types of PL have now been proposed (Figure 1): type I – neat liquids consisting of porous molecular hosts; type II – porous molecular hosts dissolved in a cavity‐excluded solvent; type III – porous media, typically frameworks, dispersed in a cavity‐excluded solvent; and type IV – porous meltable frameworks.^[3]^ Fast forward a few years to 2015 and the first porous liquids were reported, both type I and type II.[^4^, ^5^] Since then, there has been rapid growth in the development of PLs using a wide variety of porous materials as the pore carrier, including porous organic cages (POCs), metal organic cages (MOCs), zeolitic imidazolate frameworks (ZIFs), metal organic frameworks (MOFs), and covalent organic frameworks (COFs).^[6]^ However, while a significant amount of research has gone into the discovery of new PLs, there are very few studies which have investigated the uptake and subsequent controlled release of gas molecules from within the PL. Typically, this is achieved by temperature or pressure swings,[^7^, ^8^] sonication,^[9]^ or chemical displacement by the addition of a competitive guest molecule.^[10]^ However, an alternative approach is to look towards stimuli responsive porous materials, which can undergo a structural transformation in response to an external stimulus, which can include temperature, pH, magnetic fields, and light.^[11]^ However, some porous materials are not stable to dramatic changes in temperature or pH, such as POCs and COFs formed using reversible chemistries, and MOCs and MOFs formed through coordination bonds. Hence, we look towards light‐responsive materials, which incorporate a photoswitchable molecule into the makeup of the material. In this concept review, in the context of the handful of reported photoresponsive PLs, we discuss what we believe to be the important considerations that need to be taken into account when designing a photoresponsive PL, such as the choice of photoswitch and its properties, the choice of porous materials and the method used to translate it into the liquid state, and the method of incorporation of the photoswitch into the selected porous material, before discussing potential strategies to realise further examples of different types of photoresponsive PLs.

## Design Factors for Incorporating Photoswitches into Porous Liquids

**Figure 2:**
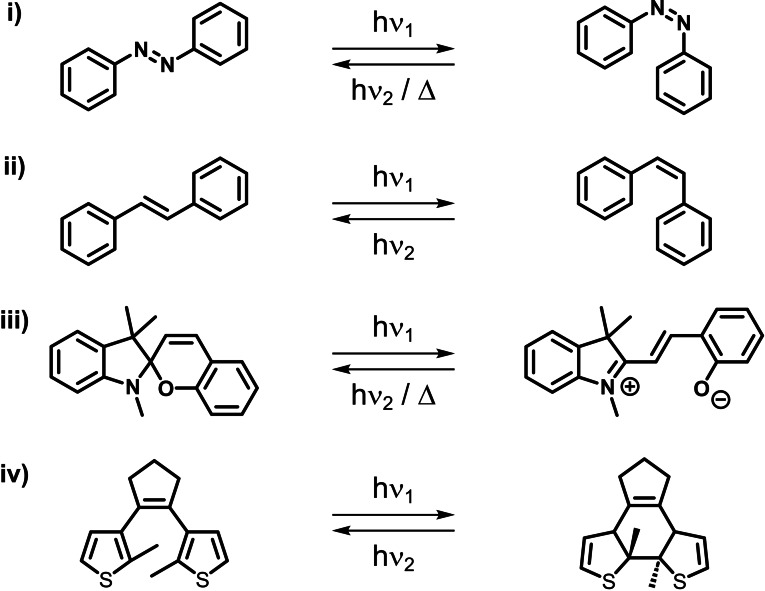
Examples of photoswitchable compounds: (i) *E*–*Z* photo/thermal isomerisation of azoarenes; (ii) *E*–*Z* photoisomerisation of stilbenes; (iii) photo/thermal cyclisation of spiropyran−merocyanines; (iv) photocyclisation of dithienylethenes.

Photoswitches are compounds that exhibit reversible structural changes upon light irradiation. Examples include azoarenes, stilbenes, spiropyrans and dithienylethenes (Figure 2).^[12]^ Photoswitches have gained considerable interest within materials science owing to their differing structural changes and physicochemical properties upon isomerisation, with applications spanning photoresponsive organic cages,[^13^, ^14^, ^15^] MOFs,^[16]^ ionic liquids (ILs)[^17^, ^18^] and liquid crystals.^[19]^ Indeed, such property changes have recently been exploited to generate photoresponsive PLs.[^20^, ^21^]

**Figure 3:**
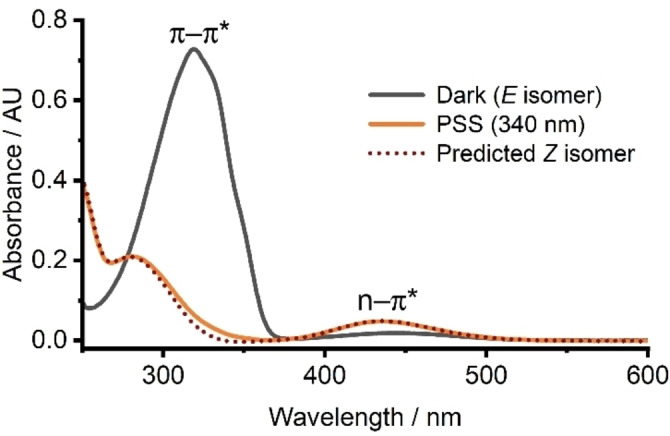
UV‐vis spectra of azobenzene in chloroform before/after irradiation at 340 nm.^[30]^ The spectrum for the predicted pure *Z* isomer is determined using the PSS calculated by the Fischer method.^[31]^

The UV‐vis spectra of photoswitches show a characteristic change in absorption upon irradiation. Figure 3 shows a representative example for azobenzene. The difference in UV‐vis absorption profiles of both states of the switch allows for preferential photoswitching under different irradiation wavelengths. Analysis of such spectra enables the determination of various photoswitching properties: the maximum absorption wavelength (λ_max_) for key absorption bands for both switch states; the photostationary state (PSS), which indicates the distribution of switch states under *in situ* irradiation; and the half‐life (*t*_1/2_) of thermal conversion of the metastable switch states to the stable form over time (where relevant). The thermal half‐life, *t*_1/2_, can vary dramatically depending on the photoswitch in question, ranging from picoseconds to years or longer. In addition, the quantum yield of photoisomerisation indicates the efficiency of the switch (per photon of light), while the fatigue resistance demonstrates its robustness to multiple switching cycles without photodegradation.

The specific design strategies for photoswitches are dependent on the application. For photoresponsive PLs, a high PSS is ideal for maximal response, as well as a half‐life of hours or longer to allow time for adsorption/desorption, and good fatigue resistance. A multitude of photoswitches have been synthesised, offering a diverse library of properties to guide PL design.[^22^, ^23^, ^24^, ^25^, ^26^] The structural change, and change in physicochemical properties, of photoswitches upon irradiation is also critical to generating a photoresponse in PLs. For example, the configurational changes in azoarenes and stilbenes can generate a change in the morphology of the material or alter the accessibility of pores.[^27^, ^28^] Different photoswitches display a range of structural and/or physicochemical changes upon switching, which may be exploited for photoresponsive PLs, such as the large change in dipole moment on photoswitching azoarenes and spiropyrans, accompanied by a drop in p*K*_a_ with spiropyrans,^[29]^ or the breaking of conjugation with dithienylethenes.

Despite their potential, the field of photoresponsive porous liquids remains underexplored. This may be due to the notable challenges in incorporating photoswitches into bulk materials, particularly in the case of framework materials where their structural rigidity often inhibits photoswitching.^[32]^ Frameworks with sufficient flexibility or free volume should be selected to accommodate isomerisation.^[16]^ Dithienylethenes offer some advantage here, since their relatively small change in free volume is more amenable to switching under structural constraint. Light penetration in bulk materials is also limited, potentially only permitting localised switching at the surface, thereby minimising any photoresponse. However, PLs can be stirred, which could lead to more of the bulk porous material being exposed to irradiation. To take one example, in a type III PL containing a suspended framework, a larger proportion of the surface area can be exposed to irradiation, which is further pronounced with decreasing particle size. In addition, metal coordination presents a unique challenge across PLs that incorporate photoresponsive MOCs and MOFs due to the potential for competing photophysical and photochemical events, such as metal‐ligand charge transfer (MLCT).^[33]^ In cases where MLCT occurs, the non‐specific excitation of isomers from irradiation of MLCT absorption bands results in unidirectional conversion to the thermodynamically favoured state. MLCT bands are typically broad and intense, therefore charge transfer events often outcompete direct photoconversion, preventing switching to the metastable state.^[34]^ Strategies to avoid MLCT include selection of metal ions less prone to charge transfer, and separation of the metal from the electron acceptor. For example, in the case of azoarenes, coordination at a remote position relative to the azo bond.^[34]^

**Figure 4:**
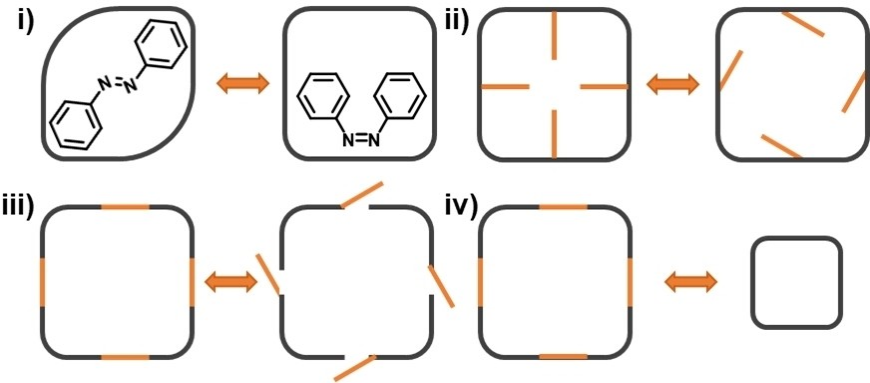
Representation of the different methods to integrate a photoresponsive moiety into a porous material: (i) loading with a photoactive guest; (ii) decorating with photoresponsive groups causing the pore size to change; (iii) decorating with photoresponsive groups to introduce a gating mechanism; (iv) incorporation of photoresponsive groups into the scaffold itself to induce a change in size and/or shape.

Once a photoswitch has been selected, there are several methods that allow photoresponsive molecules to be incorporated into porous materials (Figure 4).^[35]^ The first involves the addition of a photoresponsive guest molecule (Figure 4i). In most cases of this method, a known porous material is loaded with a photoactive guest, causing a physical property change of the host material, which can result in a change in cavity dimensions leading to varying gas uptake depending on the orientation of the photoswitchable molecule.[^27^, ^36^] Another method of incorporating a photoresponsive moiety is by decorating the porous material with photoresponsive groups (Figure 4ii/iii). This strategy utilises photoresponsive functional groups that are not key to the structure of the material, but rather are bound in or out of the material. Alternatively, such groups can be used to create photoresponsive ‘windows’ within the material, working as a gating mechanism either by opening or closing the window, preventing molecules from entering or exiting the pore. This strategy should have minimal effect in relation to a structural change but could result in the pore volume increasing or decreasing, or altering accessibility to the pore.[^27^, ^37^] A third method which could be utilised is by incorporating photoresponsive molecules into the central scaffolding components of the material itself (Figure 4iv). This could induce a structural change in the material resulting in a different size and/or shape.^[38]^

## Photoresponsive Porous Liquids

**Figure 5:**
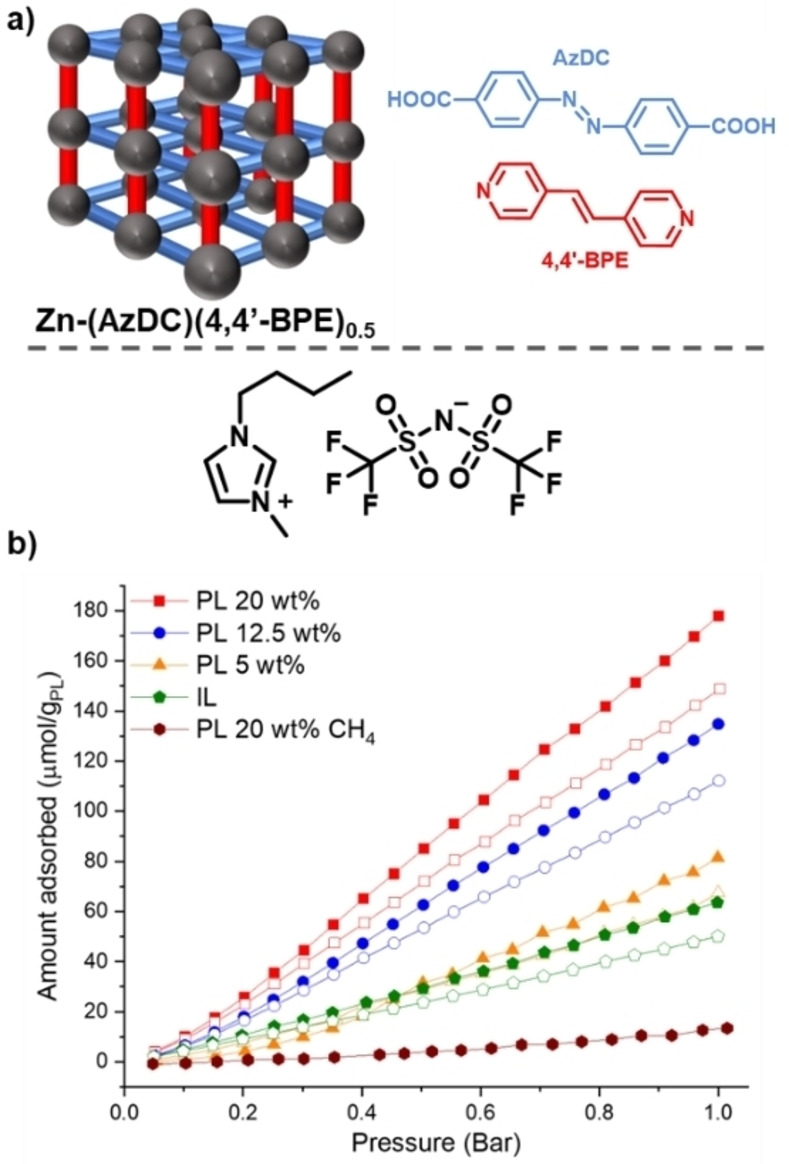
a) Illustration of the components used to form a photoresponsive Type III porous liquid from the MOF Zn(AzDC)(4,4‘‐BPE)_0.5_ and the ionic liquid [BMIM][NTf_2_]; b) CO_2_ adsorption isotherms for Zn(AzDC)(4,4‘‐BPE)_0.5_ [BMIM][NTf_2_] porous liquids and neat [BMIM][NTf_2_] ionic liquid under ambient conditions (filled) and UV light (empty). Reproduced and adapted under terms of the CC‐BY license from reference [20] Copyright (2022), with permission from Wiley‐VCH GmbH.

**Figure 6:**
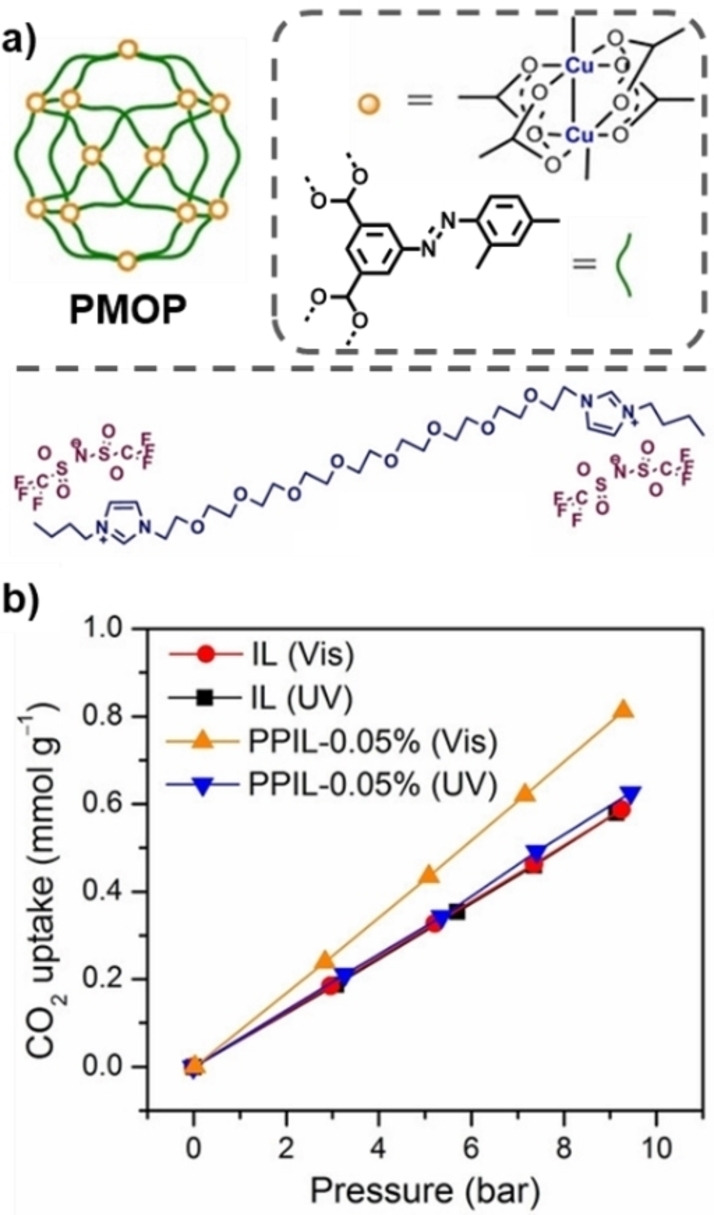
a) Illustration of the components used in the preparation of a type II photoresponsive porous ionic liquid (PPIL), with the organic moieties used in PMOP; b) High pressure CO_2_ gas sorption isotherm under visible (450 nm) and UV light (365 nm) of a 0.05 % PPIL and IL. Reproduced and adapted under terms of the CC‐BY license from reference [21] Copyright (2022), with permission from Wiley‐VCH GmbH.

**Figure 7:**
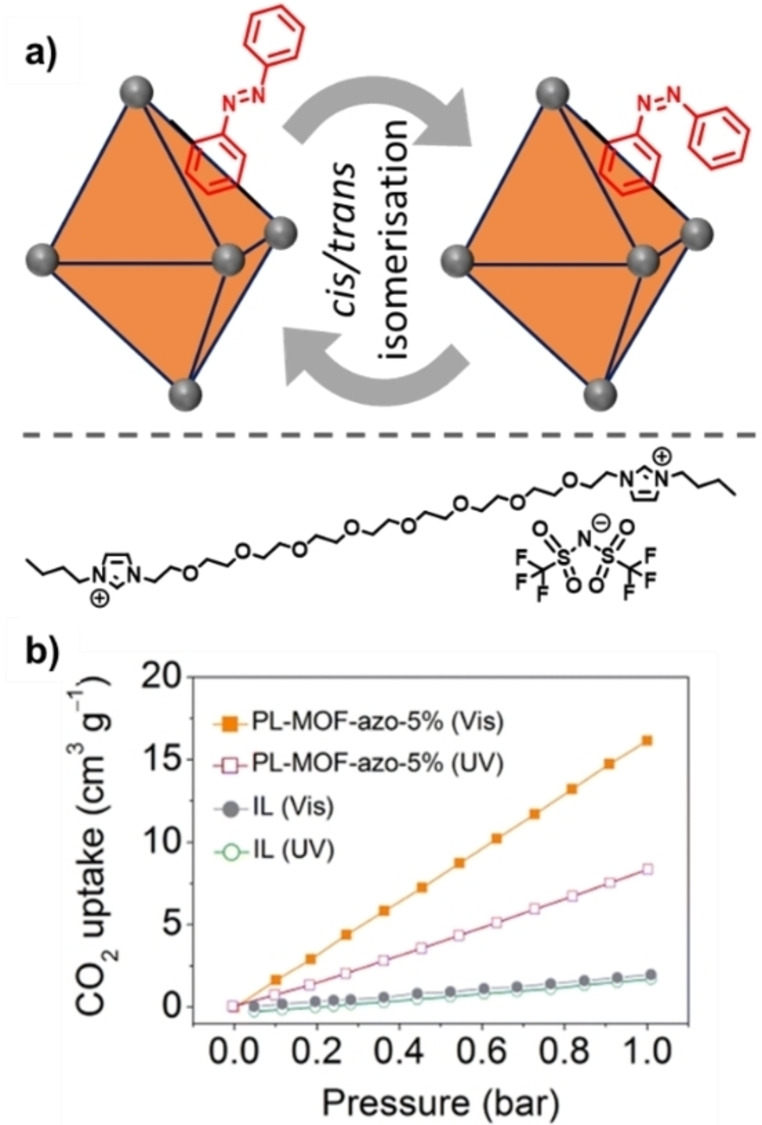
a) Illustration of the materials used in the preparation of a type III photoresponsive PL – azo‐UiO‐66 MOF with size‐excluded IL[NTf_2_]; b) CO_2_ adsorption isotherms for the neat IL (IL[NTf_2_]) and a 5 wt.% dispersion of the photoresponsive PL. Reproduced and adapted with permission from reference [39] with permission from the Royal Society of Chemistry.

To date and to the best of our knowledge, there are only three examples of PLs with photoresponsive aspects, the first two of which were reported within days of each other. Brand *et al*. reported a type III PL based on a photoresponsive MOF dispersed in a cavity‐excluded ionic liquid (Figure 5a),^[20]^ while Dinker *et al*. reported a type II system formed by dissolving a photoresponsive MOC (referred to as a photoresponsive metal‐organic polyhedron (PMOP)) in an ionic liquid (Figure 6a).^[21]^ More recently, the same research group which reported the type II PL also reported a subsequent type III PL, which used a similar strategy to that of the first type III system by dispersing a photoresponsive MOF in a bulky ionic liquid (Figure 7a).^[39]^ All three of these systems therefore incorporated metal coordination and the pore carrier contained the photoswitchable component, which were azobenzene derivatives. However, while all of the reported systems incorporated the photoresponsive groups into the scaffold itself, the type II and latter type III system were more like gated systems (*i. e*., Figure 4iii), while the initial type III PL was designed to induce a change in shape or size (*i. e*., Figure 4iv).

In the type III photoresponsive PL reported by Brand *et al*., a previously reported mixed‐linker MOF made from two well‐known photochromic molecules, azobenzene and 1,2‐bis(4‐pyridyl)ethylene, with zinc metal nodes was used.[^38^, ^40^] This MOF has also demonstrated the ability to undergo dynamic photoswitching in the solid state, creating a dynamic change in CO_2_ uptake when exposed to UV irradiation. On dispersing in a suitable size‐excluded ionic liquid to form a flowable PL dispersion (up to 20 wt% MOF), the CO_2_ uptake of the system was studied both with and without UV irradiation – on irradiating the PL samples with UV light, there was a decrease in CO_2_ capacity of up to 17 %, which was reproducible over multiple switching cycles. Photoswitching was then explored as a gas release mechanism – gas was loaded into the PL before triggering release with either UV irradiation or previously reported conventional methods (heating, sonication, or chemical displacement). Overall, UV irradiation had the highest working capacity and was a more selective and controllable release mechanism, especially in comparison to thermal release (81 % vs 61 % difference in the quantity of gas released compared to the neat ionic liquid, respectively). Additionally, the CH_4_ uptake in this photoresponsive PL was found to be minimal, giving an excellent CO_2_/CH_4_ selectivity, making it one of the most selective PLs to date.

The type II photoresponsive PL reported by Dinker *et al*. also used a previously reported MOC,^[41]^ which consisted of copper nodes and symmetrical carboxylic acid linkers with azobenzene moieties facing away from the cage, creating a MOC decorated with photoswitchable periphery functionalisation. While solubility proved to be problematic, the photoswitchable MOC could be dissolved at low concentrations in a synthesised size‐excluded ionic liquid (up to 0.2 wt%). As might be expected from the low concentration, the measured increase in CO_2_ capacity was quite low compared to the neat ionic liquid, which the authors suggest is due to aggregation of the MOP. However, a lower CO_2_ uptake was observed on exposure to UV light, for example, 0.05 wt% showed a decrease from 107 μmol/g to 87 μmol/g, an 18 % decrease, at 1 bar and 25 °C. At a higher pressure of 10 bar and 25 °C, a higher CO_2_ uptake was observed as expected, but sample irradiation was found to have a significant impact on the samples, where the 0.05 wt% sample was able to decrease its CO_2_ capacity by 30 %, 0.1 wt% decreased by 22 %, and 0.2 wt% decreased by the least at 8 %, which the authors suggest is due to the lower concentration samples having “better optical activity upon light irradiation”. As such, it would seem this is an effect of better light penetration, with fewer molecules to irradiate in the lower concentration samples, leading to more switching.

Finally, the most recent report of a photoresponsive PL was also produced by the same group as the initial type II system, but instead focused on the development of a type III photoresponsive PL.^[39]^ Here they used a MOF, azo‐UiO‐66, a derivative of arguably one of the most researched framework materials, composed of zirconium oxide nodes and terephthalic acid ligands.^[42]^ To incorporate a photoswitch into the structure, a modification to terephthalic acid was performed to incorporate diazene functionality, resulting in the formation of (*E*)‐2‐(phenyldiazenyl) terephthalic acid. When combined with ZrCl_4_, the formation of the UiO‐66 derivative is possible and has been previously reported as azo‐UiO‐66 – this MOF has shown good CO_2_ adsorption with dynamic photoswitching capabilities in the solid state.^[43]^ To incorporate the material into a photoresponsive PL, Li *et al*. then combined azo‐UiO‐66 with the same IL previously designed for the production of the type II photoresponsive PL described above, forming a type III system (Figure 7a). Similar to their previous type II report, molecular dynamics simulations were used which suggested that the IL does not enter the cavity of porous media over the lifetime of the experiments. To study the gas sorption properties of the material, CO_2_ sorption isotherms were measured on the PLs at three different concentrations of MOF (1, 2, and 5 wt.%), where the higher wt.% resulted in a higher CO_2_ capacity of 16.0 cm^3^/g at 1 bar under visible light (420 nm irradiation) (Figure 7b). The PL also showed a significant decrease in CO_2_ uptake when the sample was irradiated with UV light (365 nm) – a total of 9.0 cm^3^/g, a percentage decrease of 43 %, whereas the lower concentration PLs exhibited a lower overall CO_2_ uptake and a smaller percentage decrease upon irradiation. Interestingly, the opposite trend is seen in the previous type II photoresponsive PL study, where samples of a lower wt.% resulted in a higher percentage decrease in CO_2_ capacity upon irradiation with UV light.

## Strategies to Access Further Photoresponsive Porous Liquids

As demonstrated by the two reported photoresponsive PLs to date, by taking inspiration from photoresponsive porous solids and combining them with strategies commonly used to translate porosity from the solid to liquid state in porous liquids, there is the potential to realise further examples of photoresponsive PLs. For example, discrete porous molecules such as POCs, MOCs,^[44]^ and cyclodextrins,^[45]^ have been used to form both type I and type II PLs by incorporating functionalisation to reduce the melting point or to increase solubility in cavity‐excluded solvents, and, separately, photoresponsive derivatives of each of these species are also known.[^10^, ^46^, ^47^] In relation to forming type III PLs, simply dispersing known insoluble porous materials in cavity‐excluded solvents is a frequently used strategy in the literature for non‐photoresponsive materials, especially with ZIFs as the pore carrier. Therefore, further examples of photoresponsive type III PLs could be formed by dispersing known photoresponsive MOFs,[^16^, ^48^] ZIFs,[^32^, ^49^] and COFs[^50^, ^51^] in cavity‐excluded solvents. In addition, the reported examples of photoresponsive porous liquids all utilise size‐excluded ionic liquids, and while these are ideal from a porous liquid point‐of‐view in relation to having zero or near‐zero vapor pressures for gas uptake measurements, there are many examples of type II and III PLs that are formed with other size‐excluded solvents and oils that could be investigated with different photoresponsive materials.

Alternatively, rather than using a photoresponsive pore carrier, there is the potential to use a photoresponsive liquid as the cavity‐excluded solvent, for example, photoresponsive ionic liquids are known,^[17]^ and ILs are commonly used to make PLs, especially type III systems, so the use of photoresponsive bulky ILs with a porous material may be one alternative strategy to accessing photoresponsive type III PLs. This approach could also potentially be exploited as a method to control the cavity‐exclusion of the solvent, based on either size‐exclusion or favourable host:guest binding, and therefore acting as a triggerable gas release mechanism by active displacement from the cavities. While there are many examples of photoresponsive host:guest chemistry, to take one example, the reversible binding of an azobenzene in a cyclodextrin based system has been reported,^[52]^ and as mentioned above, cyclodextrins have been modified to make a neat type I PL.^[45]^ Finally, taking inspiration from photoresponsive ILs and type IV PLs, one could also investigate responsive coordination frameworks, for example, a reversible transformation between a discrete IL and extended coordination polymer by application of light and heat has been studied previously,^[53]^ and perhaps such a transition would have the capability to switch the gas uptake and overall porosity of the system.

Whatever strategy is adopted to form photoresponsive PLs, it is important to emphasise the requirement to fully understand the photoswitching behaviour of the porous material to ensure that it fits the criteria of the design, whether that is a prolonged half‐life or chemical stability, to ensure it will remain functionally useful when incorporated into a PL. When developing the system into a porous liquid, some of the photoresponsive characteristics may change, for example, the half‐life, which can be influenced by the solvent system being used. To some extent this has been observed in all three examples to date, where the neat porous solid and the PL demonstrate different percentage changes in gas capacity depending on the state of the material. In some ways it can be envisaged that incorporating the material into a PL would result in a more uniform response, opposed to just irradiation of the surface of the solid material, leading to an even distribution throughout the liquid. With this, it is also therefore clearly important to understand the formation of the PL, alongside the porosity, whether that is the pore carrier or fluidisation component, both before and after incorporation.

## Summary and Outlook

As discussed in this concept review, the surface of what is possible with photoresponsive porous liquids has only just been scratched, with plenty of exciting and wonderful possibilities yet to come to fruition. Inspiration for these new materials can be taken from many different areas, where elements of learning from both photoswitch and porous material design could be combined to develop new strategies towards new photoresponsive PLs. Combining these strategies could lead to the possibility of having selective mechanisms for guest uptake and release, including that of gas molecules as shown in the handful of current examples of photoresponsive PLs, and changes in the materials molecular structure could also result in on/off selectivity for the uptake of gases. There is also the potential for synergistic gas release methods, for example, a temperature‐swing could also induce reverse‐switching of certain photoresponsive molecules, leading to improved working capacities, but these would need to work together rather than cancel each other out. Light‐responsive physical properties may also be beneficial for certain applications, for example, photorheological fluids with light‐responsive viscosity having been reported.^[54]^ Some type II PLs are known to form gels after a period of time, which could allow for the study of similar materials, utilising and exploiting the control of sol‐gel transformations using photoresponse, as previously studied for ionogels.^[55]^ We hope that this short concept review shines the light on photoresponsive porous liquids, creating interest within the scientific community and we look forward to seeing new discoveries within this field.

## Conflict of interests

The authors declare no conflict of interest.

1

## Biographical Information

*Dr Michael Brand is a postdoctoral research associate at the University of Liverpool and Leverhulme Research Centre for Functional Materials Design working with Prof. Andy Cooper FRS, and carried out his PhD studies working in the same group and under the co‐supervision of Dr Becky Greenaway. His research focuses on the development of photoresponsive porous organic cages, porous liquids, and exploring new states and properties of organic cages*.

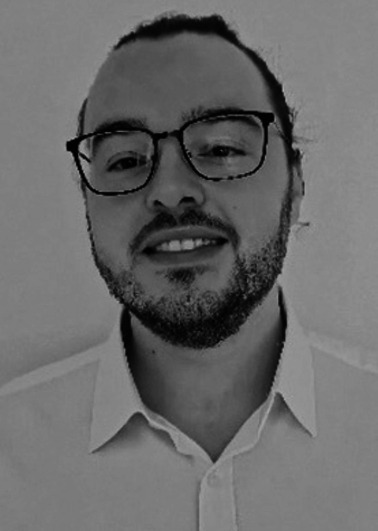

## Biographical Information

*Mr Hamish Trowell is a PhD student at Imperial College London with the React CDT. He is working with Prof. Matthew Fuchter, co‐supervised by Dr Becky Greenaway, on photoresponsive co‐ordination frameworks, liquid crystals, and DNA assembly. His research incorporates (TD)DFT for reaction optimisation and prediction of photoswitch properties. Before his PhD, he completed his undergraduate studies and MSci degree at Trinity College, Cambridge*.

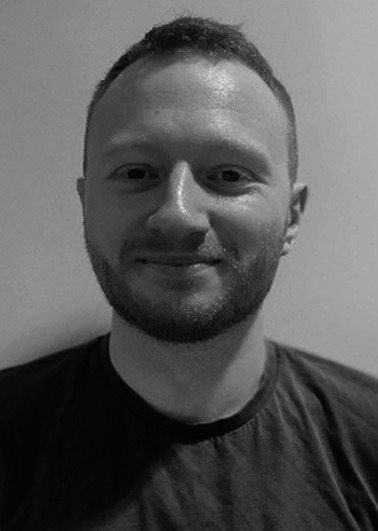

## Biographical Information

*Prof. Matthew Fuchter is Professor of Chemistry at Imperial College London. His broad research portfolio has a focus on the development of functional molecules and materials for a wide range of applications, including drug discovery, energy storage and optoelectronics. His group have a particular interest in heteroaromatic azoarene photoswitches, building from their discovery of the arylazopyrazoles in 2014. Ongoing targets for light‐addressable systems include new discovery approaches in photopharmacology, switchable phase change materials, and new photoswitchable porous materials*.

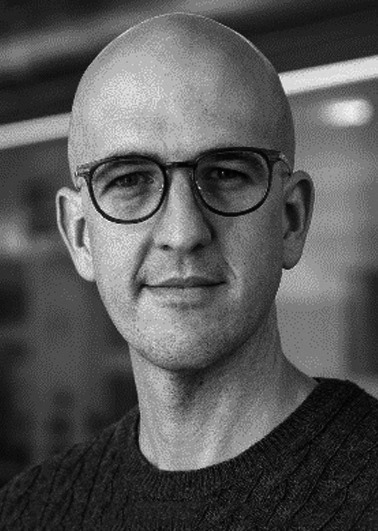

## Biographical Information

*Dr Rebecca Greenaway is a Senior Lecturer and Royal Society University Research Fellow at the Department of Chemistry at Imperial College London. Becky carried out her DPhil with Prof. Ed Anderson at the University of Oxford, before joining the University of Liverpool as a postdoctoral researcher with Prof. Andy Cooper FRS. She began her independent research in 2019 and joined Imperial College in 2020. Her research focuses on the accelerated discovery of molecular organic materials assembled using dynamic covalent strategies. This includes the investigation of non‐conventional phases of porous materials including porous liquids, and the development of high‐throughput automated workflows*.

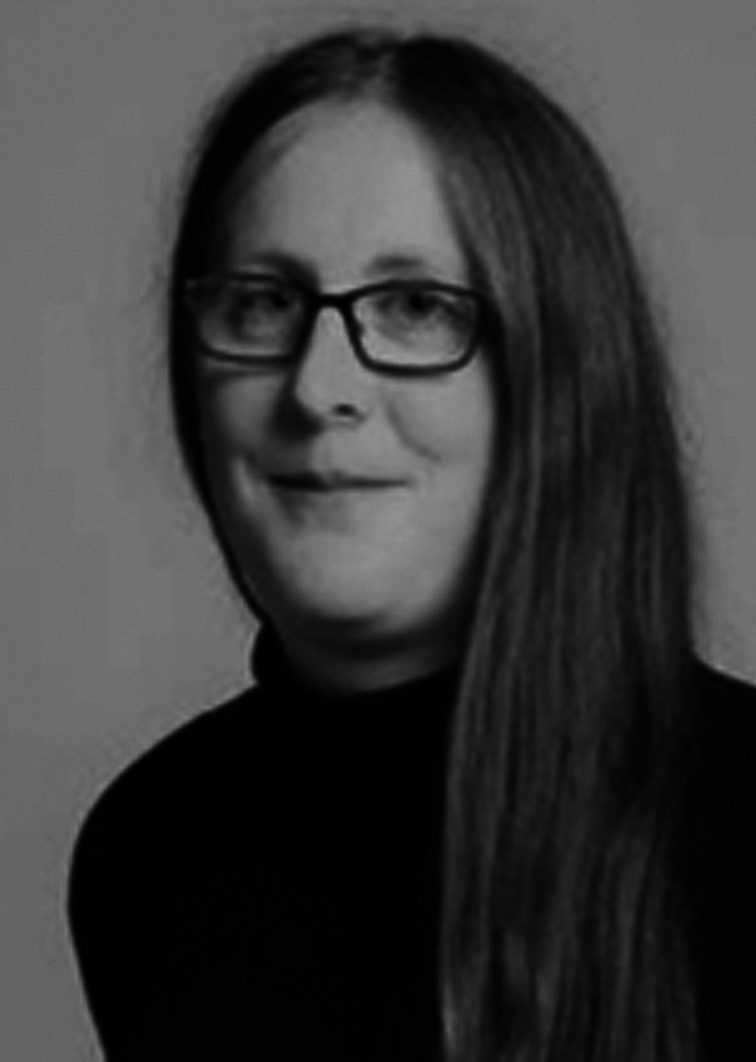

## Data Availability

Data available in article reference.
